# Evaluating the Impact of a Novel Competency-Based Education Workshop on Interns’ Perceived Preparedness in Providing Generalist End-of-Life Palliative Care

**DOI:** 10.1089/pmr.2024.0095

**Published:** 2025-06-26

**Authors:** Nessa Keane, Riana Minogue, Maeve Brassil, Dympna Waldron, Eileen Mannion, Julien O’Riordan, Orla Mongan, Sharon Beatty

**Affiliations:** ^1^ Department of Palliative Medicine, Galway University Hospital, Galway, Ireland.; ^2^ University of Galway, Galway, Ireland.

**Keywords:** competency-based, education, end-of-life care, generalized palliative care, junior doctors

## Abstract

**Objectives:**

Palliative Care provision is a key competency for all physicians. Junior doctors are actively involved in the delivery of end-of-life care in hospitals despite often feeling unprepared to do so. There has been a recent shift toward competency-based education in palliative care. The primary aim of this study was to assess the impact of a novel competency-based workshop on interns’ perceived preparedness in providing generalist palliative care at end of life.

**Methods:**

A novel competency-based education workshop was designed by a multidisciplinary team including a medicine for the elderly consultant, the director of the intern training program, the hospital end of life coordinator, junior doctors and a palliative care consultant. The workshop consists of five interactive sessions delivered to interns over a three-week period by a member of the multidisciplinary team. The Palliative Care Competence Framework Questionnaire was administered pre and post completion of the workshop to examine the impact of the workshop on attitudes, behaviors, and knowledge.

**Results:**

Prior to the intervention, 86% (n = 38) of participants reported feeling inadequately prepared to manage death and dying to the level required in their role. Overall, there was a significantly positive improvement in attitudes (*p* = 0.0314), behavior (*p* ≤ 0.0001), and knowledge (*p* ≤ 0.0001) following the competency-based workshop among participants who completed the pre- and post-intervention questionnaires.

**Conclusions:**

The findings from this study suggest a novel competency-based workshop improved interns’ perceived preparedness in providing generalist palliative care at end of life. Future initiatives will focus on validating the findings of this study.

## Introduction 

The specialty of Palliative Medicine encompasses the support of patients with myriad illness trajectories in the in-hospital, hospice and community settings. One important aspect of Palliative Medicine pertains to End-of-Life care. Frequently, in the Irish health care setting, medical practitioners from outside of Palliative Medicine, e.g., non-consultant hospital doctors and general practitioners, are involved in the delivery of end-of-life care. In recent years, there has been increased emphasis on Competency-Based Medical Education (CBME) following the observation that junior doctors undergoing traditional didactic education may be unprepared for aspects of their clinical duties.^1^ The aim of CBME is to train doctors to be safe and proficient in undertaking clinical duties based on local needs and it follows that adopting this approach in the area of Palliative Medicine will improve the ability of doctors outside of the speciality to meet the end-of-life care needs of patients.

Demand for palliative care has increased in recent years and will continue to expand in decades to come.^2^ The projected expansion for specialist palliative care in Ireland reflects demographic changes at a population level with an increase in the absolute numbers of older adults dying in addition to increased numbers of patients living longer with malignancy and chronic disease with associated specialist palliative care needs.^3,4^

The acute hospital remains the most common place of death globally.^5,6^ In Ireland, 44% of deaths occur in hospitals each year.^7^ Junior doctors are actively involved in the delivery of end-of-life care in hospitals despite feeling unprepared and unsupported to do so. Linane et al. investigated the psychological impact of end-of-life care on junior doctors, finding 11.8% screened positively for posttraumatic stress disorder based on experiences with death and dying. Challenges facing junior doctors include lack of knowledge and preparedness, difficulty communicating with family members, lack of support and feelings of failure. The study concluded that junior doctors are regularly faced with delivering end of life care, resulting in high levels of emotional distress and improved training recommended.^8^

The recognition of palliative care as a ‘core competency’ for junior doctors is well supported and recognizes the importance of training in clinical assessment, communication, multidisciplinary teamwork and prescribing to prepare junior doctors for provision of safe and effective end of life care for patients and a framework for self-care.^9^ The importance of palliative care education is recognized by the World Health Organization and the European Association for Palliative Care (EAPC).^10,11^ Lack of such education has previously been identified as a barrier to palliative care integration in health care systems across Europe.^12^ Despite this, studies have shown medical graduates report inadequate palliative care teaching and training on an international level.^13–15^

There has been a shift toward competency-based education in palliative care. *The Palliative Care Competence Framework (PCCF)*, published by the Irish Health Service Executive in 2014, details core and discipline-specific competences for generalist and specialist palliative care. The *PCCF* describes six core competencies that include (1) Principles of palliative care; (2) Communication; (3) Optimizing comfort and quality of life; (4) Care planning and collaborative practice; (5) Loss, grief, and bereavement; and (6) Professional and ethical practice in the context of Palliative Care. The Palliative Care Competence Framework was developed using the Tuning Approach which provided flexibility and autonomy to develop both core and discipline specific competences for generalist and specialist palliative care. The outcome is a clear framework for evidence-based, safe and effective palliative care for generalist and specialist practitioners irrespective of place of practice.^16^ A survey instrument based on the PCCF was subsequently developed and has shown validity and reliability.^17^

The primary aim of this study was to assess the impact of a novel competency-based workshop on interns’ perceived preparedness in providing generalist end of life palliative care. The secondary aim was to utilize the results to guide future workshop development, inform curricula and tailor undergraduate clinical exposure to palliative care.

## Materials and Methods

### Ethical approval

Ethical approval was obtained from the Galway University Hospital Research and Ethics Committee.

### Study participants

All Interns employed at a tertiary teaching hospital were eligible to participate in this study. Informed consent was obtained via *Survey-Monkey*. Support was available to participants throughout the program.

### Design of the education workshop

The education workshop entitled “When your patient is dying” is a university-affiliated interactive lecture series designed by a multidisciplinary team including a medicine for the elderly consultant, the director of the intern training program, the hospital end-of-life coordinator, junior doctors, and a palliative care consultant. The curriculum was developed using the *PCCF* specific to junior doctors and the EAPC recommendations on Palliative Care Education.^10,15^

The workshop consists of five individual sessions delivered over a three-week period by a member of the multidisciplinary team. Each workshop is focused around a number of clinical scenarios addressing core topics: (1) Death and Dying; (2) Communication; (3) Symptom Control; (4) Legal Aspects of End-Of-Life Care; and (5) Self-Care and Resilience. Each individual session addressed a single core topic.

Sessions utilize a “Flipped Classroom Approach” in which participants are provided with learning material prior to the in-person session, through PowerPoint slides, an NCHD handbook, and practice guides online. During each in-person session of the workshop, a number of clinical scenarios are described. Following presentation of each scenario, participants are given the opportunity to provide anonymous feedback on how they might handle the scenario using live audience polling via *Mentimeter.* Anonymous feedback is available in real time encouraging informal discussion among participants and with the educator. It simultaneously allows the educators to assess participants understanding and knowledge so that the subsequent content can be tailored to the needs of participants. Use of anonymous feedback on sensitive topics can encourage engagement and acknowledgment of difficulty when discussing a complex end of life care issue. Discussion is followed by didactic teaching and summaries of key points. The “Flipped Classroom Approach” of teaching improves student engagement in critical thinking.^18^

### Baseline characteristics and previous experience

Demographic details, information on current specialty post, and extent of undergraduate education and clinical experience of palliative medicine were recorded.

### Study questionnaire

Participants were invited to anonymously complete the “*Palliative Care Competence Framework Questionnaire”* both prior to and following completion of the education workshop to assess their self-reported preparedness to practice. This novel assessment tool to measure a physician’s self-reported competence was described by Connolly et al. in 2018. Based on the *PCCF*, the questionnaire was developed to assess three subscales; attitudes, behavior, and knowledge. The questionnaire consists of 27 items in total—6 items assessing attitude, 11 items assessing behavior, and 10 items assessing knowledge. Each item is rated using a 5-point Likert scale with a score of 1 consistent with a negative response, and a score of 5 consistent with a positive response. The scores for each item in a subscale are pooled and the mean or median calculated to provide an overall outcome for attitudes, behaviors, and knowledge.^17^

### Feedback from participants

Upon completion of the workshop, participants were asked open-ended questions about their experience of the workshop.

### Statistical analysis

All data collected were anonymized. Statistics were performed using GraphPad Prism version10.2.3. Survey responses are reported using descriptive statistics. Mann–Whitney (nonparametric) test for continuous variables assessed pre-to-post course changes in subscales. Significance was set at *p* < 0.05.

## Results

### Baseline characteristics

Of 53 interns who participated in the Death and Dying workshop, 44 completed the questionnaire. The median age of participants was 25 years (range 23–39 years). A spectrum of subspecialties in both medicine and surgery were represented (Table 1).

**Table 1.** Specialties

Medicine (*n* = 21)	*n*	Surgery (*n* = 23)	*n*
Not specified	5	Urology	4
Geriatric Medicine	4	Not specified	4
Endocrinology	3	Ear, nose and throat	3
Respiratory	1	Lower Gastrointestinal	3
Rheumatology	1	Upper Gastrointestinal	2
Acute Medical Unit	1	Breast	2
Radiation Oncology	1	Orthopedic	1
Medical Oncology	1	Vascular	1
General Practice	1	Cardiothoracic	1
Infectious Diseases	1	Anesthetics	1
Ophthalmology	1	Plastic	1
Cardiology	1		

### Extent of undergraduate education around palliative care principles

While 93% (n = 40) of participants reported completion of formal undergraduate teaching in Palliative Care, only 16% (n = 7) completed an undergraduate rotation through Palliative Care. Furthermore, while over half of participants (58%, n = 25) reported exposure to death since commencing Internship, 86% (n = 38) reported a perception of inadequate preparedness to manage death and dying to the level required of them in their current role (Table 2).

**Table 2.** Experience of Participants

		% (*n*)
Undergraduate Experience	Completion of an undergraduate rotation in Palliative Care	16% (7)
	Completion of Formal undergraduate teaching in Palliative Care	93% (40)
Preparedness	Perceived inadequate preparedness to manage death and dying to the level currently required in their role	86% (38)
Clinical Exposure	Exposure to Death since commencing Internship	58% (25)

### Changes in attitude, behavior, and knowledge

Twenty-two participants completed the post workshop questionnaire. eight responses were excluded from final analysis due to the omission of a significant amount of data, leaving 14 valid responses. Comparing pre-to-post course questionnaires, a significant improvement was seen across all subscales. There was a significantly positive improvement in attitudes (median/mean±SD pre 5/4.4 ± 0.8 and post 5/4.6 ± 0.7, *p* = 0.03), behavior (median/mean±SD pre 3/3.16 ± 0.9 and post 4/3.5 ± 1, *p* ≤ 0.001), and knowledge (median/mean±SD pre 3/3.2 ± 0.9 and post 4/3.7 ± 0.9, *p* ≤ 0.001) among participants (Table 3).

**Table 3.** Pre-Course and Post-Course Self-Reported Competencies in Attitudes, Behaviors, and Knowledge

Subscale	Items	Pre workshop questionnaire median (mean ± SD)	Post workshop questionnaire median (mean ± SD)	*p* value
Attitudes	1. Continued professional development and learning in relation to palliative care is part of my role 2. Being aware of the scope of and benefits of specialist palliative care in achieving a good death is part of my role 3. Providing empathetic care to people with life-limiting conditions and their families is part of my role 4. Recognizing the impact of a life-limiting condition on the person and his or her family, and being able to provide support to help the individual adapt to the changes in his/her condition is part of my role 5. Engaging with palliative care research is part of my role 6. Commitment to developing self-care strategies is part of my role	5 (4.4 ± 0.8)	5 (4.6 ± 0.7)	0.0314
Behavior	1. Communicate diagnosis and likely prognosis in an accurate and compassionate manner, taking account of the person’s needs and wishes 2. Enlist the skills of the MDT to support communication with the person with a life-limiting condition and their family 3. Recognize, address, and mediate conflict in decision-making in the context of palliative care provision 4. Provide leadership in inter-and intradisciplinary team communication in the context of palliative care provision 5. Recognize transition points in the trajectory of a patient’s illness where the goals of care change 6. Facilitate key events in the care of a patient with a life-limiting illness, e.g., family meetings, advance care planning 7. Support family members in what to expect where death is imminent 8. Provide expert leadership and guidance in palliative care practice to colleagues when requested 9. Seek and respect patients’ preferences regarding their care at the end of life, e.g., place of care, advance care plans 10. Appropriately apply the Irish Medical Council guidelines on end-of-life care in addressing ethical issues which may arise when caring for the patient with a life-limiting illness 11. Provide education in palliative care in the context of the local care environment	3 (3.16 ± 0.9)	4 (3.5 ± 1)	<0.0001
Knowledge	1. How to anticipate and respond to the needs of people with life-limiting conditions and their families in a proactive and timely manner 2. Knowledge of appropriate timing for the utilization of a palliative care approach 3. Knowledge of how to recognize and understand common trajectories of life-limiting conditions, including prognostic factors and symptoms 4. Knowledge of how to recognize and manage potentially reversible causes of clinical deterioration, as appropriate, and weigh up the appropriateness of investigations and treatments for individual patients 5. How to assess and manage distressing but uncomplicated symptoms experienced by people with life-limiting conditions 6. How to assess and manage distressing and complex symptoms experienced by people with life-limiting conditions 7. Knowledge of how to verify and pronounce death 8. Knowledge of the role of the coroner and when deaths must be reported 9. Understanding of grief, and the normal range of responses to loss 10. How to recognize risk factors for complicated grief	3 (3.2 ± 0.9)	4 (3.7 ± 0.9)	<0.0001

Likert-Scale: 1 strongly disagree; 2 somewhat disagree; 3 neutral/no opinion; 4 somewhat agree; 5 strongly agree. *p* ≤ 0.05.

### Feedback from participants

Fourteen participants completed the anonymous feedback questionnaire; 100% (14) of participants would recommend the workshop to colleagues; 93% (13) feel the workshop will influence their future practice.

Participants indicated particularly helpful aspects of the workshop were recognizing the signs of dying and prescription of anticipatory medications. One participant suggested the workshop could be improved by spacing out the sessions over a longer period of time due to the emotional nature of the topic. Relevant qualitative comments are detailed in Table 4.

**Table 4.** Participants’ Qualitative Comments

Which aspect of the workshop did you find most helpful?	It gifted a wider view on dealing with the acuity of events of patients dying and the role of palliative care
	How to manage symptoms toward the end of life
	Very intern-oriented
	How to care for people around death—I experienced this as the best way to learn
	How to declare death and clarity on the role of the coroner. Greater familiarity with Palliative Care medications
	Administrative aspects—who should fill out which forms etc. intern or registrar for example
What do you think could be improved about the workshop?	Shifting those lectures to be at an earlier time in the internship year
	Inclusion of more clinical scenarios
	Possibly space out over longer time period—heavy topic
	Provide a document/checklist for interns on what to do when patient dies during on call shift

## Discussion and Conclusions

This study explored the impact of a novel competency-based palliative care workshop on interns working at a tertiary hospital. Palliative medicine experience and training was variable among participants prior to completing the workshop. While over half of participants had been exposed to a dying patient, 86% feel inadequately prepared to deal with death and dying to the level which is expected of them. This data is in keeping with previously reported studies.^13–15^

The palliative care competency framework questionnaire was utilized to assess three subscales of competencies; attitudes, behavior, and knowledge. Quantitative analysis suggests a significant improvement in pre-to-post course questionnaires in all three subscales indicating the workshop had an overall positive impact on participants’ self-reported competencies. The authors acknowledge that the improvement observed in the Attitudes subscale is subtle and less likely to have a positive clinical impact. Feedback indicated participants felt the workshop would influence their future practice. The workshop is the first competency-based palliative medicine training program to the best of our knowledge. The positive findings suggest that there is scope for a standardized workshop that could be delivered nationally to address the variability of training that junior doctors receive.

Limitations of this study include a low number of participants completed the post-intervention survey which limits the conclusions reached in this article. Future initiatives will focus on improving post workshop questionnaire response to validate the findings of this study and incorporating the feedback provided by participants in future workshops.

In conclusion, this study suggests a novel competency-based workshop improved interns’ perceived preparedness in providing generalist palliative care at end of life.

## Abbreviations Used

CBME: Competency-Based Medical Education
EAPC: European Association for Palliative Care
NCHD: Non Consultant Hospital Doctor
PCCF: The Palliative Care Competence Framework
